# Atypical Epiretinal Proliferation in a Case of Tuberculosis Uveitis

**DOI:** 10.1155/crop/7580881

**Published:** 2026-07-17

**Authors:** Doğukan Cömerter, Eyüp Düzgün

**Affiliations:** ^1^ Department of Ophthalmology, Sultan Abdülhamid Han Training and Research Hospital, Istanbul, Turkey

**Keywords:** epiretinal membrane, epiretinal proliferation, lamellar hole–associated epiretinal proliferation, lamellar macular hole, serpiginous-like choroiditis, spectral domain optical coherence tomography

## Abstract

**Introduction:**

To report an unusual epiretinal proliferation (EP) case in tubercular serpiginous–like choroiditis.

**Case Presentation:**

A 30‐year‐old female patient with a history of uveitis presented with blurry vision and floaters for 5 days. The visual acuities of the right and left eyes were 7/10 and 9/10, respectively. A slit lamp examination of the right eye showed moderate anterior uveitis with anterior chamber cells of 2+ and 1+ cells in the anterior vitreous. The left eye showed a posterior synechia at the inferior half of the pupil but no cells in the anterior chamber. Mild vitritis was observed in both eyes. A fundus examination revealed two atrophic punch‐out lesions in the right eye and multiple pigmented chorioretinal scar–like lesions in the left eye. OCT showed cystoid macular edema in the right eye and a wide epiretinal proliferative membrane in the left eye. The immunocompetent patient had a family history of tuberculosis (TB) infection. The tuberculin skin test resulted in a ≥ 20‐mm induration, and the QuantiFERON‐TB Gold test was positive. The patient was started on topical and oral steroids. Additionally, anti‐TB treatment was added, leading to a favorable outcome. Following treatment, signs of panuveitis showed resolution, and there was an improvement in vision symptoms.

**Conclusions:**

To our knowledge, this may represent one of the first reported cases of EP associated with tubercular serpiginous–like choroiditis.

## 1. Introduction

Epiretinal proliferation (EP) is observed as a thickened yellowish‐pigmented cell collection on the retina. Histopathologic examinations revealed the presence of retinal glial cells and suggested that there might be a proliferation that mainly stemmed from Müller cells, which showed continuity within the inner retinal layers [1]. Compera et al. [2] suggested that EP was composed of fibroblasts and hyalocytes. It is also suggested that these nontractional membranes often play a role in the development of degenerative macular holes, which are characterized by intraretinal cavitation. Schumann et al. [3] reported the presence of a membrane that they named “atypical epiretinal tissue” in one‐third of lamellar macular hole (LMH) cases and a higher incidence of ellipsoid zone (EZ) and external limiting membrane (ELM) defects. It has been reported that 30.5%–60% of LMHs involve lamellar hole–associated epiretinal proliferation (LHEP) [1]. Recent international consensus terminology proposed by Hubschman et al. [4] further refined the optical coherence tomography (OCT)–based diagnostic criteria and classification of LMHs and associated epiretinal proliferative changes.

Epiretinal membranes (ERMs) were reported at rates of up to 40% in patients with uveitis [5]. It is also known that secondary ERM in young patients may occur due to retinal vascular diseases, inflammatory processes, and trauma. EP is accepted as a distinct clinical entity from conventional ERM. EP, unlike ERMs, does not have strong contractile properties. Therefore, it does not cause traction and mechanical distortion of the macular area as well as tortuosity of the retinal microvasculature [6, 7].

In this report, we aimed to present the findings of a patient with serpiginous‐like choroiditis (SLC) who was also found to have EP, a newly defined vitreoretinal interface abnormality.

## 2. Case Presentation

**Figure 1:**
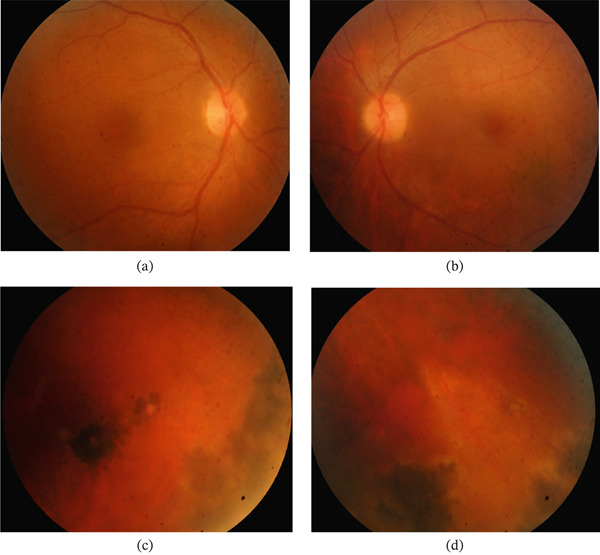
Although the color fundus photographs were blurry due to vitritis, the macular reflex in (c) the left eye was seen as faint compared with that in (a) the right eye. (b, d) Multiple atrophic punch‐out lesions in the lower quadrant and pigmented chorioretinal scar–like lesions with irregular borders in the lower quadrant of each eye.

**Figure 2:**
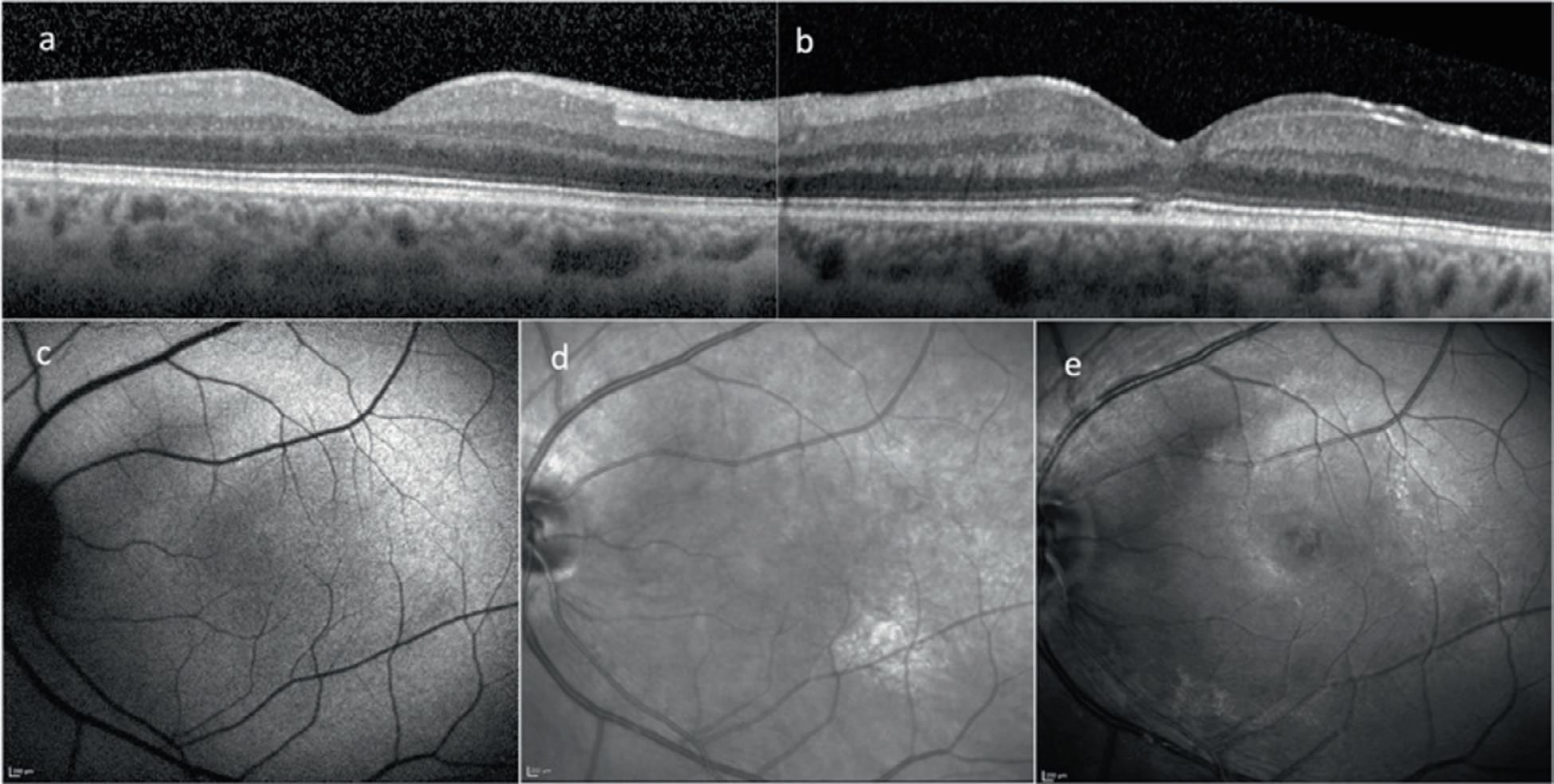
(a) OCT imaging showed a normal scan in the right eye. (b) In the temporal quadrant of the left eye, the horizontal OCT section displayed a thick epiretinal proliferative membrane with the same reflectivity as the ganglion cell layer. Mild irregularities in the ELM and EZ layers were observed without definite evidence of lamellar macular hole formation. (c) Blue autofluorescence showed loss of the macular hypoautofluorescence reflex due to EP. (d, e) On infrared and blue reflectance images, there were no peculiar findings corresponding to the area with EP.

**Figure 3:**
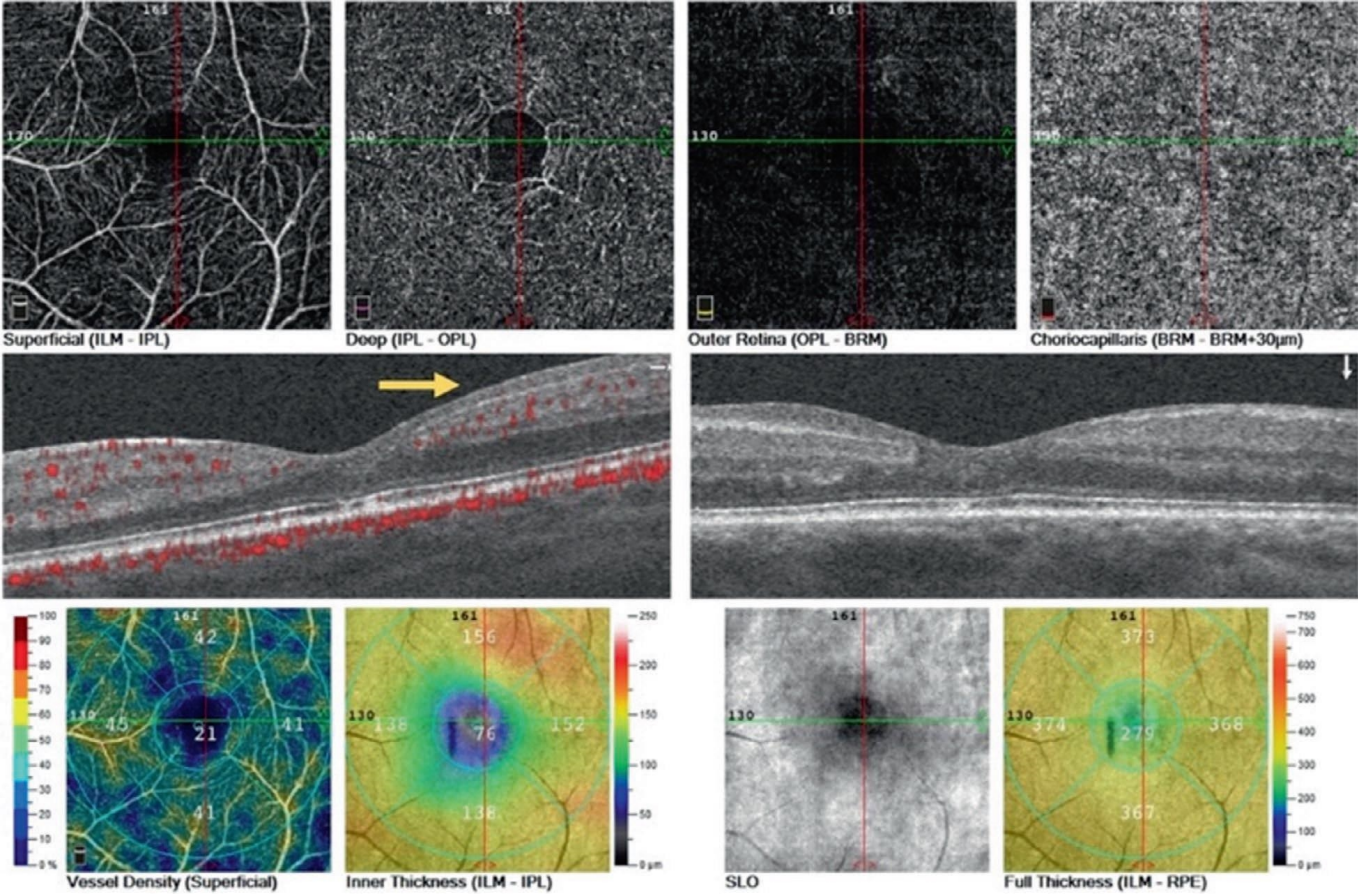
The blood flow signal was not detected in EP (yellow arrow) on the structural OCT section of the OCT‐A imaging. A nonflow area or distortion was not seen in en face images of the superficial or deep capillary plexus. There was no remarkable expansion in the foveal avascular zone area or irregularity in its circularity.

A 30‐year‐old immunocompetent woman with a history of idiopathic uveitis presented with blurred vision and floaters for 5 days. She reported having her first uveitis episode 4 years ago, with disappearing symptoms upon short‐term eye drop use and no exact diagnosis despite undergoing examinations and tests. No significant symptoms other than oral aphthous ulcers appearing a few times a year were present. The best corrected visual acuities (BCVAs) of the right and left eyes were 7/10 and 9/10, respectively, using a Snellen chart. Intraocular pressure was 15 mm Hg in both eyes. A slit lamp examination of the right eye showed moderate anterior uveitis with anterior chamber cells of 2+ and 1+ cells in the anterior vitreous. The left eye showed a posterior synechia in the inferior half of the pupil but no cells in the anterior chamber. There were no signs of keratic precipitates or iris nodules. Mild vitritis was observed in both eyes. A fundus examination showed multiple atrophic punch‐out lesions and pigmented chorioretinal scar–like lesions with irregular borders in the lower quadrant of both eyes (Figure 1). Normal autofluorescence was observed in the right eye, and macular hypoautofluorescence loss due to EP blockade was observed in the left eye. No remarkable features of EP were noted on infrared and blue reflectance imaging (Figure 2). Macular OCT showed intraretinal hyporeflective cysts in the right eye. In the left eye, we detected a wide epiretinal tissue compatible with an epiretinal proliferative membrane in the superior and temporal quadrants and minimal irregularity in the ELM and EZ layers of the foveola caused by the distorted membrane (Figure 2). The central macular thicknesses were 279 and 284 *μ*m in the right and left eyes, respectively. There were no findings of posterior vitreous detachment or LMH. Fundus fluorescein angiography (FFA) showed hyperfluorescence findings in the macula that were compatible with cystoid edema and leakage at the optic disk in the right eye. A mild leakage at the optic disk was also seen in the left eye. In both eyes, there were leakages showing a fern‐like pattern, which were more distinct in the equatorial and peripheral retina. Optical coherence tomography angiography (OCT‐A) revealed that this extra layer on the retinal surface was avascular. A blood flow signal was not detected in the thick epiretinal cell collection. No nonflow areas or distortion were seen in the superficial or deep capillary plexus (Figure 3).

Biochemical and microbiologic blood tests were all normal. We conducted venereal disease research laboratory (VDRL) tests, FTA‐ABS for syphilis, and IgM/IgG for toxoplasmosis and herpes, as well as HIV serology, which were negative, and radiologic imaging (chest CT) was performed to reveal the etiology of uveitis, which showed no signs of systemic diseases. A pathergy test also resulted negative. However, the patient had a family history of tuberculosis (TB) infection. The tuberculin skin test resulted in a ≥ 20‐mm induration, and the QuantiFERON‐TB Gold test was positive (Table 1). Pulmonology, dermatology, rheumatology, and infectious diseases departments confirmed that there were no comorbidities. The patient was diagnosed as having tubercular SLC and was given conventional anti‐TB treatment with four‐drug therapy, namely, isoniazid, rifampicin, pyrazinamide, and ethambutol, for 2 months, followed by two‐drug treatment, namely, isoniazid and rifampicin, for 4 months. Topical prednisolone acetate drops (1%) once per hour and cyclopentolate drops (1%) twice per day were prescribed. In addition to topical steroid treatment, oral methylprednisolone (1 mg/kg/day) was also added. Doses for topical and systemic steroids were gradually tapered, and then azathioprine (50 mg) tablets twice per day were initiated. The macular edema in the right eye completely disappeared after the termination of the steroid treatment. The area of the epiretinal proliferative membrane in the left eye increased over time to the superior and inferior quadrants of the macula (Figure 2). Following treatment, signs of panuveitis showed resolution, and there was an improvement in vision symptoms. The visual acuity in both eyes was 9/10 in the patient, who was followed up without an episode for 2 years through follow‐up examinations once every 3 months.

**Table 1:** Laboratory and serological results.

Parameter	Result	Reference range
Complete blood count (CBC)		
Hemoglobin (g/dL)	13.6	12.0–16.0
Hematocrit (%)	41	36–46
White blood cell count (×10^9^/L)	6.8	4.0–11.0
Neutrophils (%)	58	40–75
Lymphocytes (%)	32	20–45
Platelet count (×10^9^/L)	265	150–400
CRP (mg/L)	1	0–5
ESR (mm/h)	3.2	< 30
Renal function tests		
Blood urea nitrogen (mg/dL)	14	7–20
Creatinine (mg/dL)	0.8	0.6–1.2
eGFR (mL/min/1.73 m^2^)	> 90	> 90
Liver function tests		
AST (U/L)	22	0–35
ALT (U/L)	19	0–45
Alkaline phosphatase (U/L)	78	40–130
GGT (U/L)	20	10–45
Total bilirubin (mg/dL)	0.7	0.2–1.2
Albumin (g/dL)	4.4	3.5–5.0
Electrolytes		
Sodium (mmol/L)	139	135–145
Potassium (mmol/L)	4.2	3.5–5.1
Chloride (mmol/L)	103	98–107
Calcium (mg/dL)	9.2	8.5–10.5
Magnesium (mg/dL)	1.9	1.6–2.4
Phosphorus (mg/dL)	3.4	2.5–4.5
Serological markers		
VDRL	Negative	Nonreactive
FTA‐ABS (syphilis)	Negative	Nonreactive
Toxoplasma IgM	Negative	< 0.9 index (negative)
Toxoplasma IgG	Negative	< 10 IU/mL (negative)
Herpes simplex IgM	Negative	< 0.9 index (negative)
Herpes simplex IgG	Negative	< 1.1 index (negative)
HIV‐1/2 Ag/Ab	Negative	Nonreactive
Pathergy test	Negative	Negative
Tuberculin skin test (TST)	≥ 20‐mm induration	Positive: ≥ 10 mm
QuantiFERON‐TB Gold (IU/mL)	0.73 (positive)	Negative: < 0.35 IU/mL

## 3. Discussion

Immunohistochemical and electron microscopy studies revealed that EP tissue differed from classic ERM, which also causes LMH formation [1]. More recent OCT‐based studies also emphasized that EP represents a distinct vitreoretinal interface abnormality with unique structural characteristics and retinal layer associations [8]. Contrary to the tangential forces created by ERM due to its contractile feature, EP is claimed to cause damage to the outer layers of the retina with the vertical vitreofoveal forces it creates toward the retinal surface. For this reason, it is recommended to classify lamellar holes associated with ERM as “tractional LMHs” and holes with EP as “degenerative macular holes” [6]. In our case, posterior vitreous detachment and LMH were not detected when OCT sections were reviewed. In addition, no distinct classic ERM was observed at the vitreoretinal interface, and fine hyperreflective foci were observed on the nasal sector of the macula. These hyperreflective foci may represent early reactive vitreoretinal interface alterations rather than thickening of the internal limiting membrane (ILM). The reflectance of the epiretinal proliferative tissue in OCT sections was moderate and homogeneous and separated from the equivalent density ganglion cell layer by the nerve fiber layer with more intense reflectance. Although ELM and EZ disruptions were observed in the outer retinal layers, no interdigitation zone defect was detected. These outer retinal irregularities may theoretically represent sequelae of previous cystoid macular edema or a spontaneously healed LMH associated with reactive glial proliferation.

SLC is an immune‐mediated choriocapillaritis related to tubercular uveitis. The lesions of SLC are multifocal, sparing the juxtapapillary area, and involve the posterior pole, midperiphery, and retinal periphery. However, in rare cases, sarcoidosis, syphilis, and herpes can also lead to SLC. A positive QuantiFERON and tuberculin skin test helped establish the diagnosis of ocular TB. Patients with SLC respond well to anti‐TB therapy. Inflammatory cell infiltration in the vitreous was a remarkable feature of our patient with TB‐related SLC. Glial proliferation and membrane formation may develop as a complication of SLC due to this inflammation [9]. Although we hypothesize that chronic inflammation associated with tubercular serpiginous–like choroiditis may have contributed to EP formation through reactive Müller cell/glial proliferation, this association remains speculative. EP may also represent an incidental finding or a preexisting vitreoretinal interface abnormality that preceded the current inflammatory episode. Because prior OCT imaging before referral was unavailable, a previously existing LMH or EP cannot be completely excluded.

Metamorphopsia and visual acuity changes are the main signs indicating vitreoretinal surgery to restore visual performance in the presence of the EP. It has been reported that the visual prognosis after surgery is worse in patients with LHEP than in those without [10, 11]. It is considered a stable pathology because it does not cause wrinkling and thickening in the retina. Removing noncontractile LHEP tissue would not be as successful as the therapeutic results obtained from peeling classic tractional ERM [12]. In our case, the absence of metamorphopsia symptoms as well as the good visual acuity meant no surgical intervention was required.

## 4. Conclusion

As in the present case, we think that it would be more correct to call these membranes “atypical EP” because they are not always accompanied by a lamellar hole. To our knowledge, this may represent one of the first reported cases of EP associated with tubercular serpiginous–like choroiditis.

## Author Contributions

D.C. and E.D. wrote the main manuscript, whereas D.C. provided data collection, analysis, and conception. D.C. prepared all figures, and E.D. critically revised the manuscript.

## Funding

No funding was received for this manuscript.

## Disclosure

All authors reviewed the manuscript and agreed on the final version.

## Ethics Statement

Ethical approval is not required for this study in accordance with local or national guidelines.

## Consent

Written informed consent was obtained from the patient for publication of the details of their medical case and any accompanying images.

## Conflicts of Interest

The authors declare no conflicts of interest.

## Data Availability

The data that support the findings of this study are available from the corresponding author upon reasonable request.
